# Microaneurysms cause refractory macular edema in branch retinal vein occlusion

**DOI:** 10.1038/srep29445

**Published:** 2016-07-08

**Authors:** Taneto Tomiyasu, Yoshio Hirano, Munenori Yoshida, Norihiro Suzuki, Takeshi Nishiyama, Akiyoshi Uemura, Tsutomu Yasukawa, Yuichiro Ogura

**Affiliations:** 1 Department of Ophthalmology & Visual Science, Nagoya City University Graduate School of Medical Sciences, Nagoya, Aichi, Japan; 2 Department of Public Health, Aichi Medical University School of Medicine, Nagakute, Aichi, Japan

## Abstract

Intravitreal anti-vascular endothelial growth factor (VEGF) agents can treat macular edema (ME) in branch retinal vein occlusion (BRVO). However, refractory ME, the mechanism of which is not well elucidated, occurs frequently. Sixty-six eyes with ME secondary to BRVO were enrolled in this retrospective observational case-control study. Twenty eyes received a sub-Tenon’s capsule injection of triamcinolone acetonide (STTA), 22 eyes an intravitreal anti-VEGF injection (ranibizumab), 16 eyes were switched from STTA to ranibizumab, 4 eyes underwent vitrectomy, and 4 eyes were untreated. Multiple regression analysis and multivariate logistic regression analysis were conducted, respectively, to identify independent predictors of visual acuity (VA) prognosis and risk factors for refractory ME longer than 1 year. The mechanism of refractory ME and therapeutic approaches for identified risk factors also were investigated. Thirty-four (52%) eyes had refractory ME for over 1 year. Microaneurysms were identified as risk factors for refractory ME, leading to poor final VA. Ranibizumab suppressed microaneurysm formation and refractory ME, with early administration more effective. For already formed microaneurysms, laser photocoagulation reduced additional treatments. Microaneurysms may cause refractory ME in BRVO. Alternative therapy to suppress microaneurysms should be considered to prevent refractory ME in patients with BRVO.

Branch retinal vein occlusion (BRVO) usually results from a thrombus at the arteriovenous crossings where a thickened artery compresses the underlying venous wall, resulting in elevated venous pressure and consequent macular edema (ME), retinal ischemia, and rupture of the retinal wall with intraretinal hemorrhage^1^. In the chronic phase, after absorption of the intraretinal hemorrhage, several retinal vascular abnormalities can develop, such as capillary non-perfusion, capillary dilation, microaneurysms, telangiectatic vessels, and collateral vessels^2^. In most patients, ME is the predominant cause of visual loss in the acute and chronic phases.

The Branch Vein Occlusion Study (BVOS) group investigated the effects of grid laser treatment in patients with ME following BRVO^3^. However, the visual improvement after laser treatment occurred slowly and was limited, indicating the need for more effective treatments that provide rapid and complete visual restoration.

Elevated intraocular levels of vascular endothelial growth factor (VEGF) have been reported in patients with RVOs^4^,^5^. Intravitreal sustained release of VEGF in rabbits and primates caused vascular leakage and ME^6^. Recent prospective, randomized, and multicenter clinical trial such as the BRAVO^7^,^8^,^9^, HORIZON^10^, and RETAIN^11^ studies showed the effectiveness of anti-VEGF therapy in patients with ME secondary to BRVO. These results were far superior to that in the BVOS. Therefore, anti-VEGF therapies are now the first choice for treating ME in BRVO. However, the ME recurs frequently despite the anti-VEGF therapy, and additional injections are administered without investigating the origin of the ME. The mechanism of refractory ME has not been well studied.

In the current study, we sought to determine the risk factors for refractory ME in patients with BRVO. The mechanism and alternative therapeutic approaches also were investigated.

## Results

### Baseline characteristics

Table 1 shows the patients’ baseline characteristics. Sixty-six eyes of 66 patients (32 men, 34 women; mean age, 73 ± 8.4 years) (mean ± standard deviation [SD]) with ME secondary to BRVO were enrolled. The baseline best-corrected logarithm of the minimum angle of resolution (logMAR) visual acuity (VA) was 0.32 ± 0.31 (mean ± SD), and the baseline central retinal thickness (CRT) was 440 ± 140 (mean ± SD) μm. Twenty eyes were treated with a sub-Tenon’s capsule injection of triamcinolone acetonide (STTA), 22 eyes with an intravitreal injection of an anti-VEGF agent (ranibizumab, Lucentis, Genentech, Inc., South San Francisco, California, USA), 16 eyes were switched from STTA to an anti-VEGF agent, 4 eyes with vitrectomy, and 4 eyes were untreated. Laser photocoagulation for the non-perfusion area and/or retinal neovascularization was performed in 35 (53%) eyes.

Regarding the ocular findings associated with BRVO, a non-perfusion area larger than 5 disc diameters was observed in 23 (35%) eyes, superficial capillary telangiectasias in 30 (45%) eyes, deep capillary telangiectasias in 40 (61%) eyes, microaneurysms in 46 (70%) eyes, and collateral vessels in 56 (85%) eyes.

### Factors prognostic of the final VA

Age, gender, diabetes mellitus, hypertension, BRVO subtype, baseline VA, baseline CRT, size of non-perfusion areas, microaneurysms, collateral vessels, time of initial treatment, treatment type, and laser photocoagulation were analyzed as factors predictive of the final VA. Univariate regression analysis found that the baseline VA (*P* < 0.001; β, 0.59), refractory ME (*P* = 0.001; β, 0.28), older age (*P* = 0.002; β, 0.02), and major BRVO (*P* = 0.031; β, 0.19) (Table 2) were factors correlated with the final VA. Multivariate regression analysis of these factors detected the baseline VA (*P* < 0.001; β. 0.52 (95% confidence interval [CI] 0.29–0.75) and refractory ME (*P* = 0.019; β, 0.19 (95% CI, 0.03–0.35) (Table 2) as factors predictive of the final VA.

### Risk factors for refractory ME

Univariate logistic regression analysis detected microaneurysms (*P* < 0.001; odds ratio [OR], 18.1), older age (*P* = 0.001; OR, 1.1), collateral vessels (*P* = 0.019; OR, 12.9), and laser photocoagulation (*P* = 0.03; OR. 3.1) as risk factors for refractory ME (Table 3). Multivariate logistic regression analysis of these factors detected microaneurysms (*P* = 0.007; OR, 11.0; 95% CI. 2.25–85.9) and older age (*P* = 0.01; OR, 1.1; 95% CI, 1.04–1.27) (Table 3).

### Time of microaneurysm formation after BRVO onset

**Figure 1:**
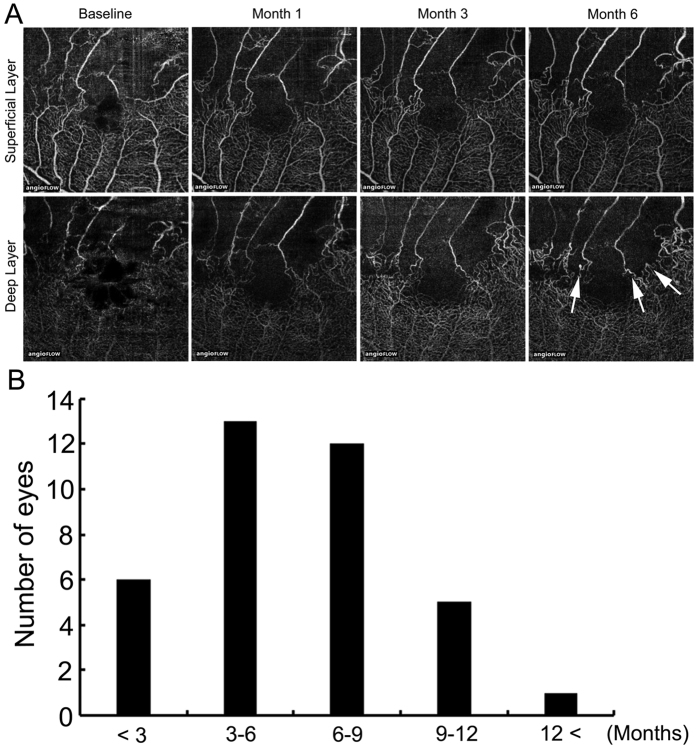
The time of microaneurysm formation in patients with macular edema in branch retinal vein occlusion. Patients, Thirty-seven eyes of 37 patients (18 men, 19 women), in whom the time of microaneurysm formation after BRVO onset was identified. (**A)** Representative optical coherence tomography (OCT) angiograms in patients with macular edema (ME) in branch retinal vein occlusion (BRVO). Top row, superficial capillary layer of the retina. Bottom row, deep capillary layer of the retina. Microaneurysms (arrows) formed on month 6. (**B)** The time of microaneurysms formation after BRVO onset. In 19 eyes (51%) of 37 eyes, microaneurysms were detected within 6 months after BRVO onset and in 31 eyes (84%) of 37 eyes within 9 months (average formation time, 6.1 ± 3.1 [mean ± standard deviation] months).

With these results, we speculated that microaneurysm formation caused refractory ME in patients with BRVO. Most microaneurysms, which seem to be a microvascular response to VEGF, originated from hypoxic retinal tissue in the inner nuclear layer and border zones^12^. In BRVO eyes, RVOs initiate retinal ischemia and ME. We could identify the time of microaneurysm formation after BRVO onset in 37 eyes of 46 eyes with microaneurysms. In 19 (51%) of 37 eyes, microaneurysms were detected within 6 months after BRVO onset and in 31 (84%) eyes within 9 months (Fig. 1B) (average formation time, 6.1 ± 3.1 [mean ± SD] months. Figure 1A shows representative optical coherence tomography (OCT) angiography images. Microaneurysms were observed on month 6.

### Microaneurysm formation, refractory ME, and VA prognosis (difference among the three treatment groups)

**Figure 2:**
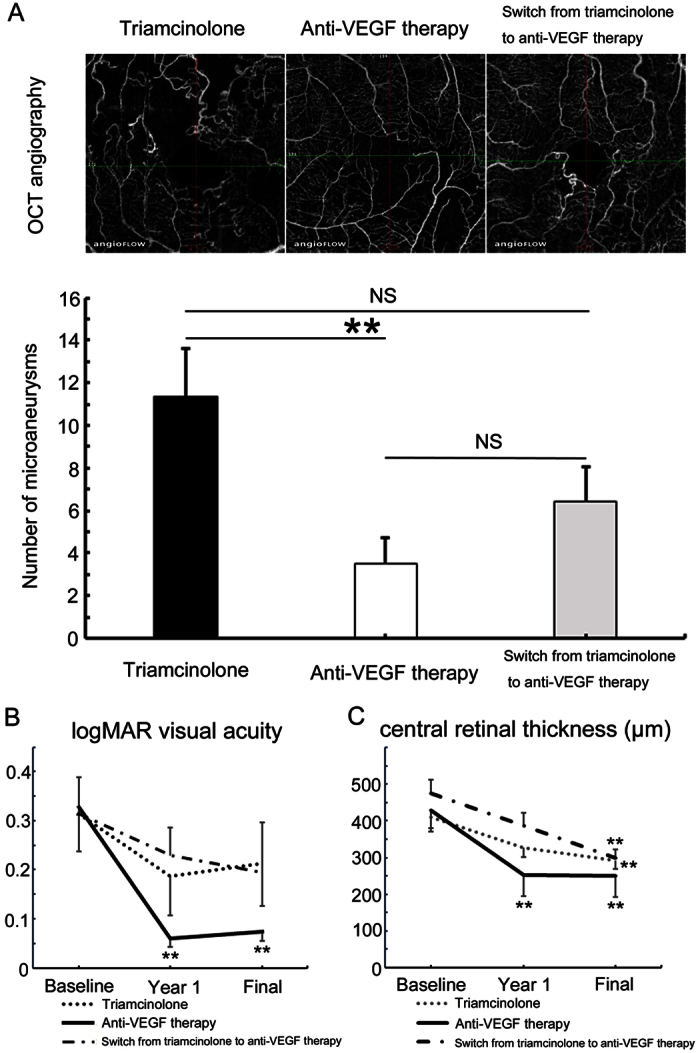
Anti-vascular endothelial growth factor therapy reduced microaneurysm formation, leading to good visual prognosis in branch retinal vein occlusion. Patients, Sub-Tenon’s capsule injection of triamcinolone acetonide (Triamcinolone): Twenty eyes of 20 patients (9 men, 11 women). Anti-VEGF therapy: Twenty-two eyes of 22 patients (13 men, 9 women). Switch from triamcinolone to anti-VEGF therapy: Sixteen eyes of 16 patients (7 men, 9 women). (**A**) The number of microaneurysms after each treatment. The panels in the top row are representative images from each treatment group. Anti-vascular endothelial growth factor (VEGF) therapy significantly (P < 0.01) reduced microaneurysms formation compared with the Triamcinolone group. Error bars represent the standard error of the mean above or below. NS, not significant. (**B**) Changes in the logarithm of minimal angle of resolution (logMAR) visual acuity (VA) in each treatment group. Anti-VEGF therapy significantly improved the log MAR VA, while triamcinolone or switch therapy did not do so. (**C**) Changes in central retinal thickness (CRT) with each treatment. The CRT significantly (*P* < 0.01) decreased with all treatments at the final visit. Only the anti-VEGF therapy significantly (*P* < 0.01) reduced the CRT at year 1. The number of injections: Triamcinolone 2.4 ± 2.2, anti-VEGF 5.2 ± 2.4, switch from triamcinolone to anti-VEGF 2.4 ± 2.0 (triamcinolone) and 3.4 ± 2.3 (anti-VEGF) (mean ± standard deviation).

Significantly (*P* < 0.01) fewer occurrence of microaneurysms was seen in eyes treated with anti-VEGF agents (41%), while in eyes treated with STTA and switch therapy from triamcinolone to anti-VEGF agents, microaneurysms were observed in 90% and 81%, respectively. Moreover, anti-VEGF agents significantly (*P* < 0.01) reduced the number of microaneurysms compared with the STTA-treated group (Fig. 2A). These results supported that over-expression of VEGF leads to microaneurysm formation.

When we checked the frequency of refractory ME associated with each treatment, in eyes treated with anti-VEGF therapy, less refractory ME was seen (18%) compared with STTA (57%) and switch therapy (81%). Regarding the VA prognosis, anti-VEGF agent-treated eyes significantly improved, while eyes treated with STTA and switch therapy did not do so (Fig. 2B). The percentage of eyes with better than 0.5 decimal VA at the final visit was 65% (13 of 20 eyes) in the STTA group, 82% (18 of 22 eyes) in the anti-VEGF group, and 69% (11 of 16 eyes) in the switch therapy group. At the final visit, the CRT in each group decreased significantly (*P* < 0.01) (Fig. 2C). However, frequently recurring ME might result in a poor final visual prognosis in eyes treated with STTA and switch therapy (Fig. 2B).

### Prompt anti-VEGF therapy reduces the number of microaneurysms and frequency of refractory ME

**Figure 3:**
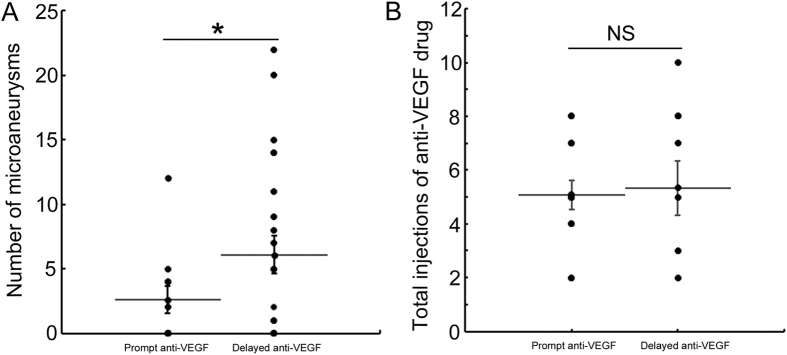
Prompt anti-vascular endothelial growth factor therapy suppressed microaneurysm formation in branch retinal vein occlusion. Patients, Prompt anti-vascular endothelial growth factor (VEGF) therapy: Fifteen eyes of 15 patients (7 men, 8 women). Delayed anti-VEGF therapy: Twenty-three eyes of 23 patients (13 men, 10 women). (**A)** The number of microaneurysms that formed after prompt or delayed therapy with anti-VEGF drugs. Prompt was defined as within 3 months and delayed as over 3 months after branch retinal vein occlusion (BRVO) onset. Prompt anti-VEGF treatment significantly (*P* < 0.05) reduced the microaneurysms formation. (**B)** The number of total injections of the anti-VEGF drug. There were no significant difference between the two groups. NS, not significant.

We compared the number of microaneurysms between prompt (within 3 months after BRVO onset) and delayed (over 3 months after BRVO onset) anti-VEGF therapy. Thirty-eight eyes (including eyes with switch therapy) were treated with intravitreal ranibizumab injections, i.e., 15 eyes with prompt anti-VEGF therapy and 23 eyes with delayed anti-VEGF therapy. Prompt anti-VEGF therapy significantly (*P* < 0.05) reduced the number of microaneurysms (Fig. 3A). The incidence rates of refractory ME were 20% with prompt anti-VEGF therapy and 65% with delayed anti-VEGF therapy (*P* < 0.01). The total number of injections in the prompt anti-VEGF therapy group was not more than that in the delayed anti-VEGF therapy group (Fig. 3B).

### Laser

**Figure 4:**
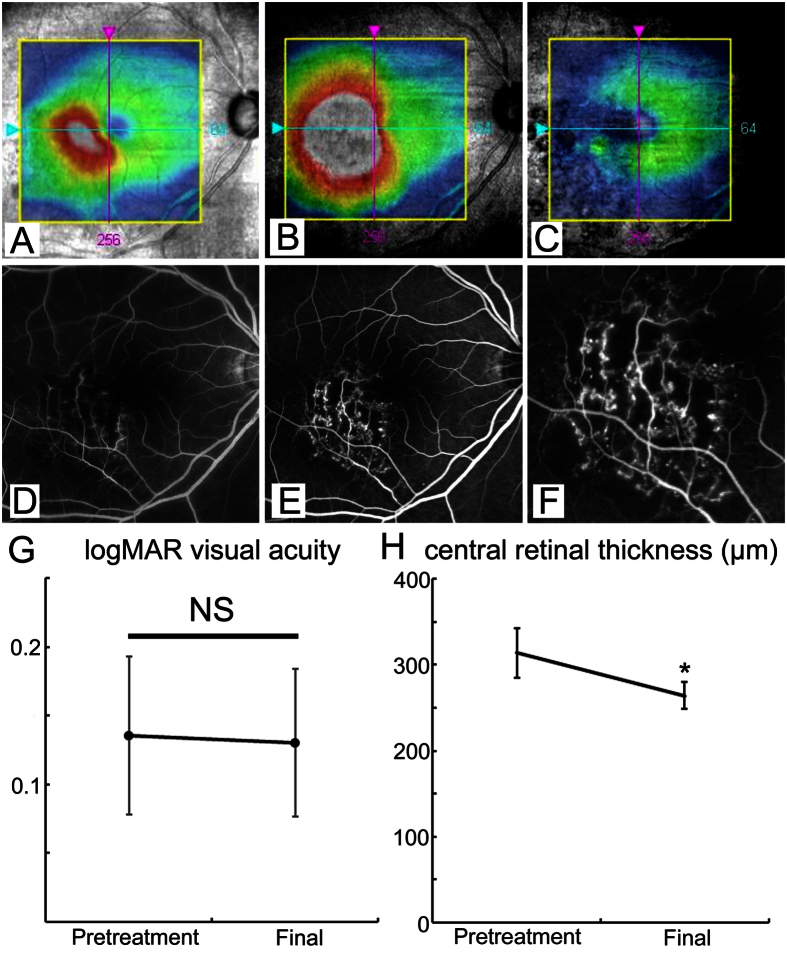
Direct laser photocoagulation targeted at microaneurysms was effective for refractory macular edema in branch retinal vein occlusion. Patients, Eleven eyes of 11 patients (5 men, 6 women). (**A–C**) optical coherence tomography (OCT) colored retinal map in patient with macular edema (ME) in branch retinal vein occlusion (BRVO). (**A**) At baseline, the visual acuity (VA) was 0.8 decimal unit. (**B**) Fourteen months after BRVO onset, the VA was 0.7. The ME worsened despite twice sub-Tenon’s capsule injection of triamcinolone. (**C**) Twenty-four months after direct laser photocoagulation for microaneurysms, the ME resolved. However, the VA remained 0.6. (**D**) Baseline fluorescein angiogram (FA) (early phase) showed that the microaneurysms were barely visible. (**E**) FA (early phase) 14 months after BRVO onset. (**F**) Magnified image of (**E**). Numerous microaneurysms were seen around the non-perfusion areas. (**G**) Changes in the logarithm of minimal angle of resolution (log MAR) VA before and after application of direct laser photocoagulation. There was no significant difference. (**H**) Changes in the central retinal thickness (CRT) before and after application of direct laser photocoagulation. The CRT significantly (*P* < 0.05) decreased after direct laser photocoagulation. Error bars represent the standard error of the mean above or below. NS, not significant.

Eleven of 34 eyes with refractory ME underwent direct laser photocoagulation at the microaneurysms. The average time after BRVO onset was 34 ± 9.8 (mean ± SD) months. The VAs after direct laser photocoagulation did not improve significantly, but the CRT decreased significantly (*P* < 0.05) (Fig. 4). Moreover, eight of 11 eyes treated with direct laser photocoagulation did not need additional treatments. Figure 4 shows a representative case. Microaneurysms were not observed at baseline, whereas numerous microaneurysms causing ME were detected 14 months after BRVO onset (Fig. 4). Direct laser photocoagulation targeting the microaneurysms resolved the ME (Fig. 4C).

## Discussion

In the current study, we found that refractory macular edema in patients with BRVO was associated with microaneurysms formation and resulted in poor final visual acuity. We also found that microaneurysms were formed at about 6 months after the disease onset and blockage of VEGF reduced the microaneurysms formation, leading to better final visual prognosis. If the anti-VEGF agents were administered earlier, the effect was more powerful. Furthermore, for already formed microaneurysms, direct laser photocoagulation was effective in reducing additional treatments.

Previous randomized multicenter studies have reported that ME in BRVO can be treated with anti-VEGF drugs^7^,^8^,^9^,^10^,^11^. However, refractory ME persists despite frequent anti-VEGF drugs injections. Once the ME becomes refractory, the final visual prognosis worsens (Table 2). The patients need frequent hospital visits with more associated costs. Therefore, detecting the risk factors for refractory ME is critical to gain an understanding of the pathology of ME in BRVO and may lead to alternative therapeutic approaches. Furthermore, if alternative therapy can reduce refractory ME, the patients might be free from the treatment burden earlier.

In the current study, we evaluated microvascular abnormalities in BRVO eyes using multimodal imaging with fundus photographs, OCT, fluorescein angiography (FA), and OCT angiography, then found that microaneurysm formation may be a risk factor for refractory ME in patients with BRVO. If so, alternative therapy targeting the microaneurysms or suppressing microaneurysm formation may reduce the treatment burden.

The other prognostic factor for the final VA was the baseline VA, which was reported previously^13^,^14^. We also analyzed the cause of poor baseline VA, but that was unclear (data not shown). Another risk factor for refractory ME was age, possibly because the endothelium of the blood vessels would be damaged as the patients age. Previous animal studies failed to exhibit persistent ME because the animals had healthy vascular systems^15^,^16^,^17^, suggesting that aging vessels are needed for ME to persist.

We have used STTA as the first-choice treatment for ME associated with BRVO until ranibizumab was approved for use in August 2013 in Japan. After approval, anti-VEGF therapy (ranibizumab) was used alternatively as a first-choice treatment in our hospital. Therefore, in the current study, several treatments were performed for ME associated with BRVO.

Interestingly, anti-VEGF therapy suppressed the number of microaneurysms compared with STTA injections. Over-expression of VEGF was reported in BRVO eyes^4^,^5^. Furthermore, it was reported that over-expressed VEGF resulted in microaneurysm formation in an animal study^18^. Blockage of VEGF might reduce microaneurysm formation due to continuous VEGF expression in BRVO eyes.

We also found the average time of microaneurysm formation after BRVO onset (Fig. 1). In diabetic retinopathies, microaneurysms were considered as early findings in the disease course. However, as we showed in the current study, microaneurysms in BRVO were not that common at the onset of BRVO and gradually formed over time after venous occlusion. The formation of microaneurysms in BRVO might differ from that in diabetic retinopathy. Briefly, venous obstruction leads to turbulent blood flow, elevated venous pressure, and overload of drainage capacity that might cause ME in the acute phase^19^. Chronic increased retinal venous blood flow velocity causes shear stress on the venous endothelium and endothelial injury^20^,^21^. High shear stress gradients and turbulent blood flow can cause reactive proliferative changes in the endothelial cells and endothelial damage^22^. In this scenario, over-expression of VEGF might cause microaneurysm formation, leading to refractory ME. Therefore, it takes time for microaneurysm to form after BRVO onset. It is clear that earlier blockage of VEGF suppressed the number of microaneurysms (Fig. 3), resulting in a lower incidence of refractory ME. Although delayed (over 3 months after BRVO onset) all treatment was unrelated to refractory ME in the current study (Table 3), we speculated that it might be, because the eyes treated with STTA within 3 months of BRVO onset also were included in the multivariate logistic regression analysis. Some cases with BRVO have resolution of ME and improvement in VA without intervention^23^. Therefore, in these cases, intervention is sometimes postponed until the VA deteriorates. However, a clinically relevant improvement beyond 20/40 was uncommon without intervention^23^. Moreover, it is unknown which cases would have resolution of ME without treatment. Early treatments with another anti-VEGF drug (bevacizumab, Avastin, Genentech Inc.) have been reported as predictive of functional and visual improvements^24^,^25^. Taken together, we think that ME in BRVO should be treated as soon as possible before microaneurysms form.

Interestingly, the total number of injections in the prompt anti-VEGF therapy group was not more than that in the delayed anti-VEGF therapy group. We speculated this occurred because earlier blockage of VEGF inhibited microaneurysm formation causing refractory ME, which might shorten the disease duration. A previous randomized multicenter study reported that the effect of delayed treatment with ranibizumab was comparable to that of prompt treatment^10^. However, the improvements in VA were six and two letters less in the Early Treatment Diabetic Retinopathy Study VA than prompt treatment at months 12 and 24, respectively. In the HORIZON study^10^ with ranibizumab, the incidence of refractory ME was not compared between the prompt- and delayed-treatment groups. Microaneurysm formation itself might cause the difference in visual improvement.

We previously reported that microaneurysms in BRVO eyes formed at the edge of the non-perfusion area and in the collateral vessels^26^. In the current study, collateral vessel formation was associated with refractory ME in univariate logistic regression analysis (Table 3). Collateral vessels usually lead to spontaneous resolution of ME^19^. However, once microaneurysms form in the collateral vessels, they might cause refractory ME, suggesting that collateral vessels might have an opposite effect on ME.

We also found that the microaneurysms causing refractory ME were treatable by direct laser photocoagulation. While the VA did not improve after direct laser photocoagulation, the CRT decreased and fewer treatments were needed. In the current study, direct photocoagulation was performed 34 months after BRVO onset. Sakimoto *et al*. reported that application of direct laser photocoagulation 20 months after BRVO onset improved vision in patients with chronic ME secondary to BRVO^27^. Earlier application of direct photocoagulation might improve the VA. Another benefit of direct photocoagulation was the reduction in the additional treatments. The RETAIN study^11^ reported that frequent injections of ranibizumab were still needed for unresolved ME 4 years after BRVO onset. Therefore, it is important not only for patients to have a lower treatment burden but also to have reduced medical expenses. We previously reported that indocyanine green angiography (ICGA)-guided laser photocoagulation was effective in idiopathic macular telangiectasia and diabetic ME^28^,^29^, both of which can cause ME due to microaneurysms. The same method might be applicable in patients with refractory ME in BRVO.

The current results are important to understand the pathology in ME with BRVO and detect the cause of the refractory ME. Earlier administration of anti-VEGF agents and direct photocoagulation targeting the microaneurysms might decrease the treatment burden and medical expenses.

The current study had several limitations. First, this study was a retrospective observational study and the intervention was not controlled. Second, the sample size was small. Further studies with larger samples or prospective, randomized, and multicenter clinical trials are needed to confirm these results and decrease the treatment burden.

## Methods

### Patients

The institutional Review Board of Nagoya City University Graduate School of Medical Sciences approved this single-center, retrospective, observational, and case-control study. The research methods and analysis adhered to the tenets of the Declaration of Helsinki. All patients visited Nagoya City University Hospital from March 1, 2005, through October 1, 2014, and were followed for longer than 1 year (mean follow-up period, 38 months; range, 12–125 months). All patients underwent a complete ophthalmic examination including measurement of the best-corrected VA, indirect ophthalmoscopy, fundus photography, OCT (Cirrus HD-OCT, Carl Zeiss Meditec, Jena, Germany), FA (indocyanine green angiography [ICGA]), and/or OCT angiography (RTVue XR Avanti, AngioVue, Optovue Inc., Fremont, California, USA). FA was performed using a wide-field laser ophthalmoscope Optos 200Tx and/or confocal scanning laser ophthalmoscopy (Heidelberg Retina Angiograph 2, Heidelberg Engineering, Heidelberg, Germany). Visual acuity was measured using a standard Japanese decimal chart and converted to logarithm of minimal angle of resolution (logMAR) to perform statistical analyses. Refractory ME was defined as ME which persisted or recurred over 1 year after the disease onset. Microaneurysm formation was judged using ophthalmoscopy, fundus photographs, FA, and/or OCT angiography. The counting of microaneurysms was done at one year after BRVO onset. All patients provided their past medical history including diabetes mellitus and hypertension. Patients were excluded when the captured images were inadequate for evaluation because of ocular movement or cataract as were those who had not been followed longer than 1 year.

### Intervention

STTA (20 mg/0.5 ml) (Kenacort, Bristol-Myers Squibb, Tokyo, Japan) was injected into the sub-Tenon’s capsule as a first-choice treatment for ME associated with BRVO from March 2005 through July 2013. The intravitreal anti-VEGF drug ranibizumab was used as an alternative first-choice from August 2013 and thereafter, when ranibizumab was approved for use to treat ME associated with BRVO in Japan. After that, STTA was switched to intravitreal ranibizumab injections when STTA did not result in reducing ME. Vitrectomy was performed when STTA was ineffective and before ranibizumab was approved for use. Patients who did not want treatment were observed without intervention. Additional treatments were applied for recurrent ME when the CRT exceeded 250 μm. Scatter laser photocoagulation was performed for retinal neovascularization and/or non-perfusion areas larger than 5 disc diameters detected by FA. Grid laser photocoagulation was not performed for macular edema in this current study.

### Direct laser photocoagulation for microaneurysms

FA and/or ICGA were performed before direct laser photocoagulation. Direct laser photocoagulation was applied to microaneurysms using a multicolor laser system (Novus Varia, Lumenis, Salt Lake City, UT, USA) with the following settings: wavelength, 561 nm; power, 100–200 mW; spot size, 50–100 μm; and duration, 0.02–0.05 sec. Additional laser photocoagulation was applied for residual microaneurysms causing ME.

### Statistics

Statistical analyses were performed using Project R (R Core Team 2015, Vienna, Austria) and the significance level was set at *P* < 0.05. Univariate analyses were performed to detect factors predictive of the final VA and risk factors for refractory ME, then the factors with *P* values less than 0.05 were selected for multivariate regression analyses. Post hoc Bonferroni correction was used to compare the number of microaneurysms among the three treatment groups. Fischer’s exact test was used to confirm the relation between refractory ME and prompt anti-VEGF therapy. Changes in the logMAR VA or CRT were assessed using one-way repeated-measures analysis of variance (ANOVA) and post hoc Bonferroni correction. Two-way repeated-measures ANOVA was used to investigate differences in the clinical courses of the VA and CRT among the groups. The paired *t*-test was used to compare the numbers of microaneurysms between the prompt- and delayed-treatment groups and the logMAR VA and CRT before and after direct laser photocoagulation.

### Study approval

The Institutional Review Board of Nagoya City University approved the study protocol. Each patient provided written informed consent following a detailed explanation of the procedures.

## Additional Information

**How to cite this article**: Tomiyasu, T. *et al*. Microaneurysms cause refractory macular edema in branch retinal vein occlusion. *Sci. Rep.* **6**, 29445; doi: 10.1038/srep29445 (2016).

## Figures and Tables

**Table 1:** Patient’s baseline characteristics, ocular findings, and treatments.

Age (years ± SD)	73 ± 8.4
Gender (male/female)	32/34
Diabetes mellitus (yes/no/unknown)	3/60/3
Hypertension (yes/no/unknown)	31/30/5
Subtype (major BRVO/macular BRVO)	36/30
Initial VA (logMAR) (mean ± SD)	0.32 ± 0.31
Initial CRT (μm) (mean ± SD)	440 ± 140
NPA larger than 5 disc diameters (yes/no/unknown)	23/42/1
NPA larger than 3 disc areas within arcade vessels (yes/no/unknown)	23/42/1
Superficial capillary telangiectasia (yes/no/unknown)	30/10/26
Deep capillary telangiectasia (yes/no/unknown)	40/0/26
Microaneurysms (yes/no)	46/20
Collateral vessels formation (yes/no)	56/10
Initial treatment within 3 months (yes/no)	47/19
Refractory macular edema	34/32
Treatment	
STTA	20
Anti-VEGF therapy	22
Switch from STTA to anti-VEGF therapy	16
Vitrectomy	4
No treatment	4
Retinal scatter laser photocoagulation (yes/no)	35/31
Direct laser photocoagulation (yes/no)	11/55

SD, standard deviation. BRVO, branch retinal vein occlusion, logMAR, logarithm of minimal angle of resolution. CRT, central retinal thickness. NPA, non-perfusion area. STTA, sub-tenon’s capsule injection of triamcinolone acetonide. VEGF, vascular endothelial growth factor.

**Table 2:** Univariate and multivariate regression analysis to detect factors predictive of final VA.

Univariate regression analysis		
Factor	β	*P* value
Age	0.02	0.002**
Gender	−0.01	0.927
Diabetes mellitus	0.17	0.416
Hypertension	0.06	0.510
Subtype (Major BRVO)	0.19	0.031*
Initial logMAR VA	0.59	<0.001***
Initial CRT	<0.01	0.019*
Microaneurysms	0.16	0.090
Collateral vessels	0.20	0.106
Prompt (within 3 months) all treatment	−0.04	0.709
Refractory ME	0.28	0.001**
Treatment	–	0.6963
STTA	0.21	0.298
Anti-VEGF therapy	0.07	0.722
Switch therapy	0.17	0.403
Vitrectomy	0.17	0.504
**Multivariate regression analysis**		
**Factor**	**β (95% CI)**	***P* value**
Initial logMAR VA	0.52 (0.29–0.75)	<0.001***
Refractory ME	0.19 (0.03–0.35)	0.019*
Age	0.01 (−0.0006–0.02)	0.067
Initial CRT	0.0001 (−0.0004–0.0006)	0.715
Subtype (Major BRVO)	0.09 (0.03–0.35)	0.200

BRVO, branch retinal vein occlusion. logMAR, logarithm of minimal angle of resolution. VA, visual acuity. CRT, central retinal thickness. ME, macular edema. STTA, sub-Tenon’s capsule injection of triamcinolone. VEGF, vascular endothelial growth factor. β, β coefficients. CI, confidential interval. ME, macular edema.

**Table 3:** Univariate and multivariate logistic regression analysis to detect risk factors for refractory ME.

Univariate logistic regression analysis		
Factors	OR	*P* value
Age	1.1	0.001**
Gender	0.7	0.465
Diabetes mellitus	2.0	0.580
Hypertension	0.9	0.900
Subtype	1.4	0.472
Initial logMAR	1.8	0.484
Initial CRT	1.0	0.086
NPA size	1.1	0.431
Microaneurysms	18	<0.001***
Collateral vessel formation	13	0.019*
Prompt (within 3 months) all treatment	1.1	0.880
Treatment	–	0.114
Retinal scatter laser photocoagulation	3.1	0.030*
**Multivariate logistic regression analysis**		
Factors	**OR (95% CI)**	***P* Value**
Microaneurysms	11 (2.3–86)	0.007**
Age	1.1 (1.0–76)	0.010*
Collateral vessels	3.1 (0.27–76)	0.385
Retinal scatter laser photocoagulation	2.6 (0.68–11)	0.166

logMAR, logarithm of minimal angle of resolution. CRT, central retinal thickness. NPA, non-perfusion area. OR, odds ratio. CI, confidential interval.
